# Application of neural network-based image analysis to detect sister chromatid cohesion defects

**DOI:** 10.1038/s41598-023-28742-6

**Published:** 2023-02-06

**Authors:** Daiki Ikemoto, Tomoya Taniguchi, Kouji Hirota, Kiyoshi Nishikawa, Kan Okubo, Takuya Abe

**Affiliations:** 1 Graduate School of Systems Design, Tokyo Metropolitan University, Asahigaoka 6-6, Hino-Shi, Tokyo, 192-0397 Japan; 2 Department of Chemistry, Graduate School of Science and Engineering, Tokyo Metropolitan University, Minamiosawa 1-1, Hachioji-Shi, Tokyo, 192-0397 Japan

**Keywords:** Biological techniques, Computational biology and bioinformatics

## Abstract

Sister chromatid cohesion (SCC) is mediated by the cohesin complex and its regulatory proteins. To evaluate the involvement of a protein in cohesin regulation, preparations of metaphase chromosome spreads and classifications of chromosome shapes after depletion of the target protein are commonly employed. Although this is a convenient and approved method, the evaluation and classification of each chromosome shape has to be performed manually by researchers. Therefore, this method is time consuming, and the results might be affected by the subjectivity of researchers. In this study, we developed neural network-based image recognition models to judge the positional relationship of sister chromatids, and thereby detect SCC defects. Transfer learning models based on SqueeezeNet or ResNet-18 were trained with more than 600 chromosome images labeled with the type of chromosome, which were classified according to the positional relationship between sister chromatids. The SqueezeNet-based trained model achieved a concordance rate of 73.1% with the sample answers given by a researcher. Importantly, the model successfully detected the SCC defect in the *CTF18* deficient cell line, which was used as an SCC-defective model. These results indicate that neural-network-based image recognition models are valuable tools for examining SCC defects in different genetic backgrounds.

## Introduction

Sister chromatid cohesion (SCC) is mediated by the cohesin complex, forming a ring-like structure and holding sister chromatids until the onset of mitosis. Cohesin consists of four proteins: SMC3, SMC1, RAD21, and SA1/SA2^1,2^. Because the location and structure of cohesin dynamically changes during the progression of the cell cycle, the function of cohesin is controlled by many regulatory proteins. In particular, the associators of cohesin, including PDS5A/B, Sororin, and WAPL, and the cohesin loader consisting of NIPBL and MAU2, play a significant role in cohesion establishment, maintenance, and dissolution. Although biochemical reconstitution of cohesin activity has been achieved in vitro with these essential SCC regulators^3,4^, dozens of proteins have been reported to regulate SCC directly or indirectly^2,5^. CTF18, an ATPase that constitutes an RFC-like proliferating cell nuclear antigen (PCNA) loader with CTF8, DCC1, and RFC2-5, is an evolutionarily conserved cohesin regulator^6–8^. The function of CTF18 in SCC is likely mediated through PCNA loading on the leading strand, although the detailed molecular mechanism is not well known^9^.

SCC defects are usually examined through the microscopic observation of metaphase chromosome spreads^10^. Although classification criteria differ among laboratories, SCC-defective cells tend to have more open sister chromatids. If SCC is completely compromised, sister chromatids are separated from each other. The problem with examining SCC defects using this method is that the evaluation and classification of chromosome shapes are performed manually by researchers, and the lines between each category are often blurred. Moreover, because minimally 50–100 metaphase chromosomes must be classified in each strain/sample for a quantitative analysis, this analysis requires considerable effort and time. These problems allow for human errors or the incorporation of researchers’ subjective bias. For these reasons, a machine-learning model that automatically classifies chromosome shapes is required.

Currently, image processing methods based on convolutional neural networks (CNNs) are widely used in various fields, including pathological examination, face recognition, and so on^11,12^. In the field of chromosome analysis, computational techniques have been developed for karyotype analysis, which are employed for prenatal diagnosis^13,14^. However, the application of automatic chromosome analysis, except for karyotype analysis, remains limited. In this study, we applied CNN-based image recognition models to classify the shape of chromosomes into three types, depending on the positional relationship of sister chromatids. The trained model achieved a maximum concordance rate of 73.1% with the example answers (EA) given by a researcher and successfully detected the SCC defects of *CTF18*^*-/-*^ cells. Based on these results, we propose neural network-based image recognition models as useful tools for automatically classifying the shape of chromosomes and examining SCC defects in mutant cell lines.

## Results

### Dataset preparation

**Figure 1:**
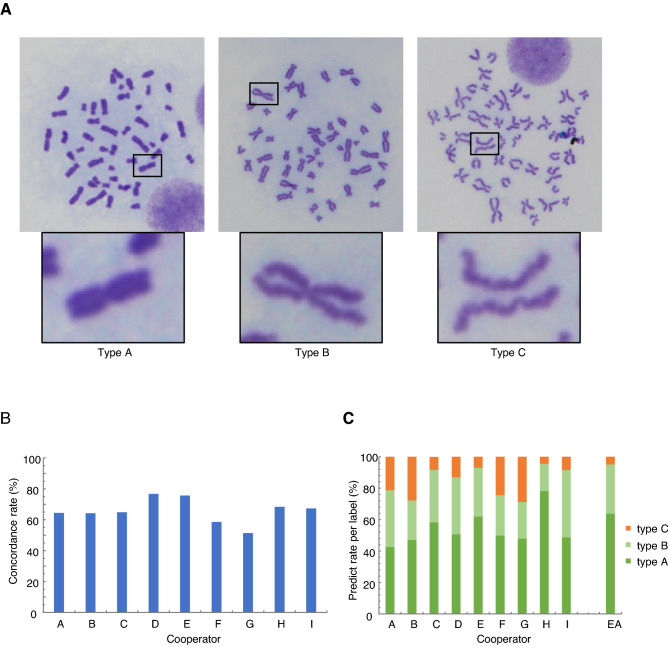
Classification of chromosome types of WT TK6 cells. (**A**) Representative images for each label. (**B**) Concordance rates between EA and answers from each cooperator. (**C**) Distribution of each type of chromosome.

In this study, we used the TK6 human B lymphoblastoid cell line, which is widely used for in vitro genotoxicity tests^15,16^. TK6 cells exhibit normal and stable karyotypes, except for their chromosome 13 trisomy^15^. Metaphase chromosome spreads were prepared from wild-type (WT) TK6 cells using a conventional method (see “Materials and Methods”). Then, 2,144 single-chromosome images that did not overlap with other chromosomes from 150 cells were cut out. Chromosome images were classified into three types (Fig. 1), and these labels were used as EA. Well-cohered tight chromosomes were classified as type A, chromosomes in which the arms were separated were classified as type B, and chromosomes in which sister chromatids were also separated at the centromere were classified as type C ^17,18^. All chromosome images from WT cells were divided into three groups: training data, validation data for model selection, and testing data for model evaluation. To divide the dataset, we first randomly selected 654 chromosome images from 43 cells as the test dataset. The distribution of types A, B, and C in this test dataset should be close to the actual distribution of each chromosome type in WT TK6 cells.

### Individual differences in classification of chromosome images

As stated above, the boundaries between each type of chromosome are not absolute, causing the classification criteria to vary between observers. To experimentally verify this, preparatory to applying CNN, the test dataset containing 654 chromosome images was manually classified by nine cooperators (referred to as cooperators A, B, C, D, E, F G, H, and I). Before classification, each cooperator was trained using several labeled chromosome images, as shown in Fig. 1A. The concordance rates between EA and each cooperator are shown in Fig. 1B. The average, maximum, and minimum concordance rates were 65.7, 76.8, and 51.4%, respectively. There was a marked difference in the rate of each chromosome type (Fig. 1C). For instance, while the rate of type C chromosomes was 4.8% in EA, it varied from 4.6 to 28.8% when the same test dataset was classified by each cooperator. This result shows that the classification criteria certainly differ among observers, and demonstrates the requirement of computational analysis, which has a fixed standard.

### Estimation model using SqueezeNet or ResNet-18

**Figure 2:**
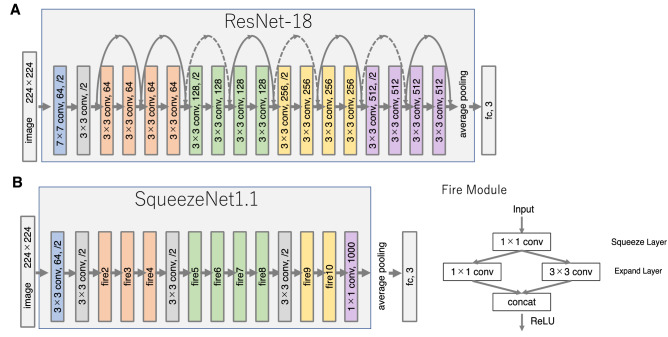
Schematic representations of transfer learning architectures. (**A**) ResNet-18. (**B**) SqueezeNet.

As pre-trained models for transfer learning, we used two different CNNs: SqueezeNet-based model and ResNet-18-based model (thereafter simply referred to as SqueezeNet and ResNet-18). Both models were trained on the ImageNet dataset to classify an image into 1,000 object categories (keyboard, mouse, pencil, many animals, etc.), and were used for an efficient image classification with a limited number of images^12,19^. ResNet was the winning model for the ILSVRC in 2015. Deepening the network is expected to improve the representation capability and classification accuracy; however, simply adding more layers is not sufficient to train it efficiently. ResNet successfully increases the depth of layers by introducing residual blocks, which combines a convolutional layer and a shortcut connection that adds the input of the previous layer directly to the layer behind it (Fig. 2A). In contrast, SqueezeNet is a lightweight model built by stacking fire modules with reduced parameters and computational complexity by utilizing 1 × 1 convolutions (Fig. 2B). The dimensionality of the input feature map is reduced by the 1 × 1 convolution of the squeeze layer, and it is restored when a feature extraction is performed by the 3 × 3 convolution of the expanded layer. However, the number of parameters is reduced by replacing some of them with a 1 × 1 convolution. The fully connected layer (fc) prior to the output of the network was trained using the chromosome dataset, and the other layers of SqueezeNet or ResNet-18 were used as fixed feature extractors.

Because the rate of type C chromosome images was much lower than that of type A or type B images in WT cells, we added 221 type C images to the dataset for a well-balanced training. From these 1,711 chromosome images, we randomly selected 400 chromosome images for validation data and 1,275 for training data in a fixed seed (Supplementary Fig. 1). All images were processed before being fed into the fully connected layer (see “Materials and Methods”). For each analysis, we repeated the validation and training data selection three times to reduce data selection bias. Moreover, with each data set, we repeated the training, validation, and testing cycles 10 times, meaning that the score obtained after each analysis was the average of 30 trials.

### Three classification of chromosome images by trained models

**Figure 3:**
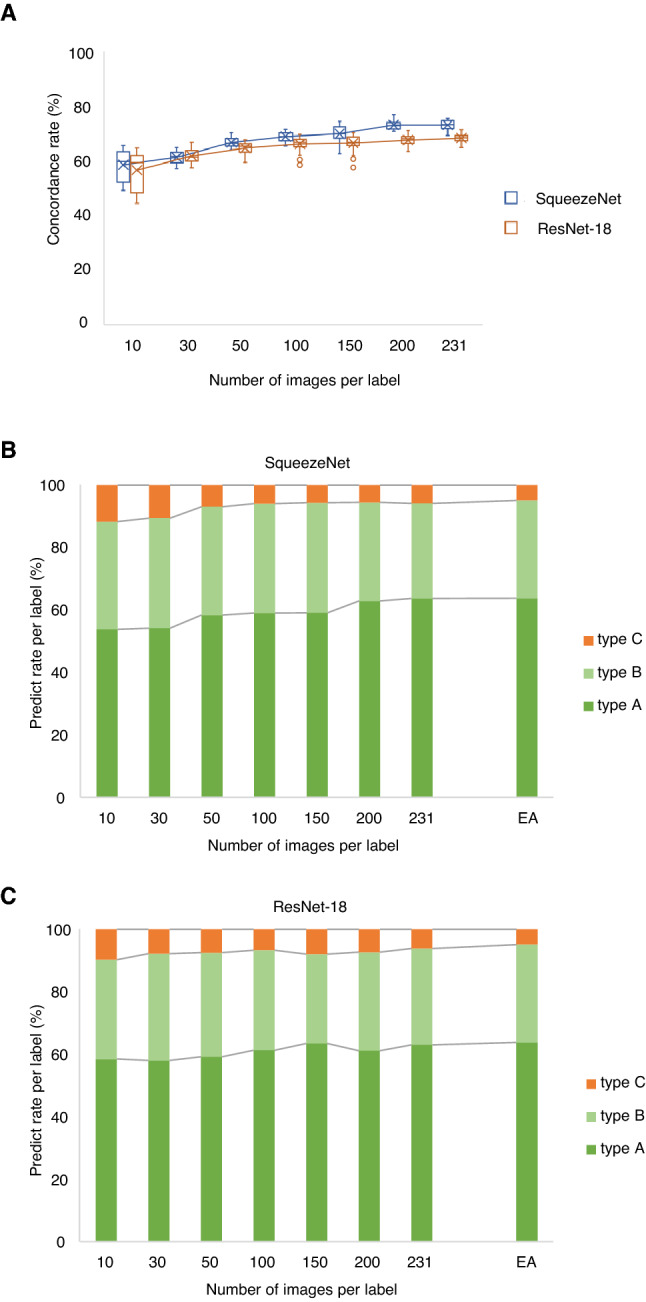
Chromosome type classifications by the trained models. (**A**) Concordance rates between EA and model’s PA for each number of training images per label. (**B** and **C**) Distribution of each type of chromosome for each number of training images per label in SqueezeNet (**B**) and in ResNet-18 (**C**).

To estimate the number of chromosome images required for training, the CNN models were first trained with different numbers of datasets, which were randomly selected from the training data and contained equal numbers of type A, type B, and type C images. After training, the models were fed 654 chromosome images as the test data. The concordance rates between EA and predicted answers (PA) from the model improved as the number of training images increased (Fig. 3A). At most of points, the SqueezeNet achieved higher concordance rates than the ResNet-18. Because the concordance rate seemed to be saturated at the training with 693 chromosome images (containing 231 of each type of chromosome), further analysis was conducted with this number of training data. Under these conditions, SqueezeNet achieved a 73.1% concordance with EA, whereas it was 68.2% in ResNet-18. Considering that the concordance rates ranged from 51.4 to 76.8% when the classification was conducted by cooperators (Fig. 1B), the concordance rates achieved by the models seem to be sufficient for practical use. Regarding the distribution of each type of chromosome, the rates of type A, type B, and type C were 63.8%, 31.4%, and 4.9% in EA; 63.7%, 30.5%, and 5.8% in SqueezeNet; and 63.0%, 30.9%, and 6.1% in ResNet-18; respectively when the models were trained with 693 chromosome images (Fig. 3B and C). These distribution patterns were quite similar, and it indicates that the classifications done by a researcher are fully reproducible by the trained models.

### Visualization of characteristic sites of chromosome

**Figure 4:**
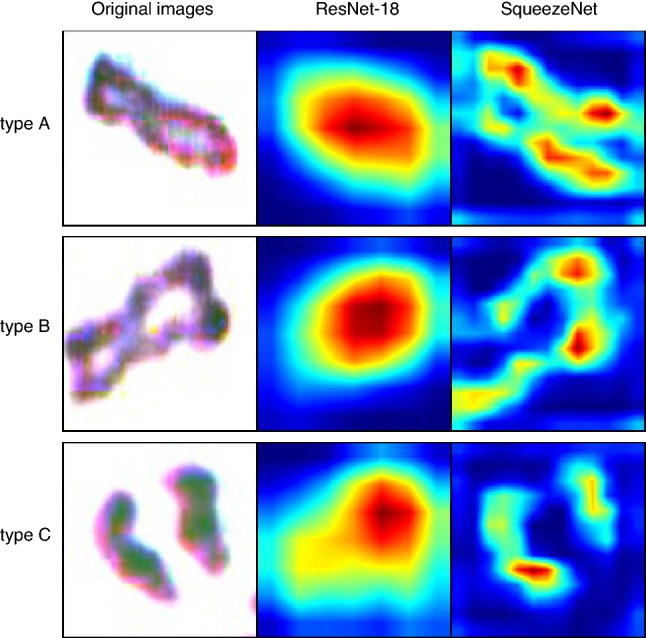
Visualization of the focus of each model with Grad-CAM.

To understand the regions of image where SqueezeNet and ResNet-18 look, we used Grad-CAM, which is commonly used to visualize the characteristic sites of images^20^. Several examples of this analysis are presented in Fig. 4. As a general trend, SqueezeNet focuses on each chromatid end, whereas ResNet-18 focuses on the centromere where sister chromatids are joined together. Because the classification should be judged by the positional relationship between each sister chromatid, the result may explain the higher concordance rates of SqueezeNet over ResNet-18.

### Establishment of a *CTF18* KO cell line as a SCC-defective model

**Figure 5:**
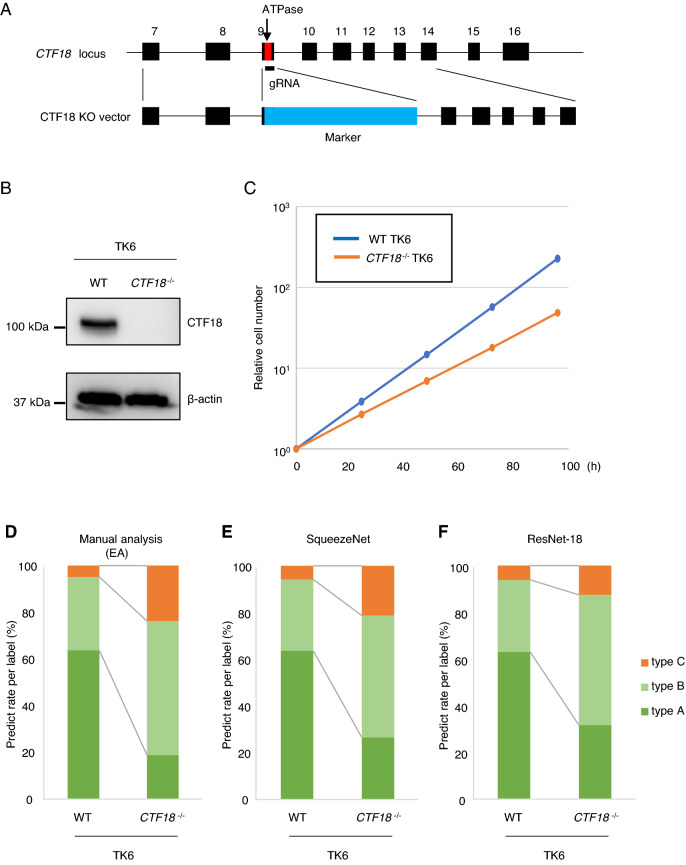
Establishment and analysis of *CTF18*^*-/-*^ TK6 cells. (**A**) Schematic representation of the *CTF18* gene locus and gene targeting knockout construct. (Closed boxes) Exons; (Marker) drug resistance genes; (Red box) the sequence encoding the ATPase domain of CTF18. (**B**) Whole-cell lysates were prepared from cells of the indicated genotypes. CTF18 and *β*-actin (loading control) were detected by Western blotting. (**C**) Growth curves of the indicated cell lines. Cells (1 × 10^5^) of the indicated genotypes were inoculated in 1 mL of medium, counted, and passaged every 24 h. Distribution of each type of chromosome in WT TK6 and *CTF18*^*-/-*^ TK6 cells. (**D**) EA. (**E**) SqeezeNet. (**F**) ResNet-18.

To confirm whether the trained model accurately detects the SCC defects of mutant cells, we chose a *CTF18* KO cell line as an SCC-defective model. This is because CTF18 is a well-approved SCC regulator, and the knockout of *CTF18* is supposed to cause relatively mild SCC defects based on previous observations^8,21,22^. To establish *CTF18*^*-/-*^ cells, we constructed gene-targeting constructs designed to delete an exon encoding the Walker A motif of CTF18, which is essential for the function of ATPase family proteins (Fig. 5A). The absence of CTF18 protein expression in the established *CTF18*^*-/-*^ cells was confirmed by a western blot analysis (Fig. 5B, Supplementary Fig. 2). *CTF18*^*-/-*^ cells were viable, but their proliferative capacity slightly decreased (Fig. 5C).

### Detection of SCC defects in *CTF18*^-/-^ cells with the trained model

To compare the rates of chromosome types in *CTF18*^*-/-*^ cells with those in WT cells, chromosome images were prepared from *CTF18*^*-/-*^ cells in the same manner as for WT cells. The rates of each type of chromosome obtained from manual analysis or from the trained models are shown in Fig. 5D–F. The concordance rates between EA and PA were a bit lower when the models classified the chromosomes of *CTF18*^*-/-*^ cells probably because these models were trained with WT TK6 chromosomes (Supplementary Fig. 3). In manual analysis, CTF18 depletion increased the rates of type B chromosomes from 30.5 to 52.2% and those of type C chromosomes from 5.8% to 21.3% (Fig. 5D), indicating that CTF18 depletion causes SCC defects in TK6 cells, as shown earlier in different cell lines and species^6,7^. In both SqueezeNet and ResNet-18, the results showed the same trend as that of the manual analysis (Fig. 5E and F). From these results, we concluded that CNN-based models are sufficiently competent to analyze the chromosomes of SCC-defective mutant cells.

## Discussion

Cohesin associators and regulators have been extensively explored for over 4 decades using biochemical and genetic approaches^23–25^. Although all essential players in SCC might have already appeared, there could be other unknown regulators whose loss partially impairs the function of cohesin, or some proteins might play roles in SCC in specific cell types.

To characterize the role of cohesin regulators by morphological approaches, classification of metaphase chromosomes has been used as an easy and reliable method^10^. In many studies, several chromosome images were shown as representative images of each category, and the rate of each category obtained by analyzing several hundred cells was shown as quantitative data^17,25–27^. However, in actual samples, many chromosomes are difficult to classify because they are located at the border of two categories. Owing to these populations, the classification criteria depends on the researcher, as shown in Fig. 2. Such populations do not cause a big problem when analyzing mutant cells deficient in cohesin subunits, cohesin loaders, or critical cohesin regulators, whose losses induce obvious and drastic chromosomal changes. However, in the case of gene mutations causing only mild SCC defects, a careful analysis is necessary to prevent subjectivity.

In this study, we examined whether CNN-based image recognition models are sufficiently sensitive to study non-essential cohesin regulators. CTF18 depletion caused the chromosome shape to be more open, but it did not induce severe SCC defects or premature sister chromatid separation in TK6 cells. Nevertheless, the model fully detected the difference in chromosome shape between WT TK6 cells and *CTF18*^*-/-*^ cells, as a researcher did. Thus, we propose the model and similar neural network-based approaches as powerful tools for examining SCC defects in non-essential cohesin regulators.

The current model achieved a maximum concordance rate of 73.1% with EA for the WT TK6 sample. This concordance rate is comparable to or slightly higher than those from the two researchers; however, in some cases, SqueezeNet yielded apparently wrong answers (Supplementary Fig. 4). To increase the accuracy, the models should be improved with several modifications in future studies. The first is to increase the labeling accuracy for both training data and test data. This is because some chromosome images are difficult to classify into a certain category, as mentioned above, and incorrect labeling may have occurred. Refining the training data by double-checking the labeling with multiple researchers or by removing data that are difficult to classify might be effective in improving the accuracy of measurement. Another improvement might be the removal of photobombed chromosome fragments. Even if the area is very small, CNN models sometimes seem to focus on them, which researchers automatically remove from the analysis. In particular, this processing seems important for analyzing type C chromosomes, which are determined by the positional relationship between two separated objects.

Currently, the model requires cropped single chromosomes that do not overlap with other chromosomes, causing the analysis to not fully avoid arbitrary choices by researchers. In the future, these chromosome analyses should be conducted using automatically captured and cropped chromosome images. Automatic capture can be performed using latest microscopes. To crop single chromosomes, CNN-based models that recognize each chromosome in a metaphase cell and automatically cut out single chromosomes can be developed. A region extraction using image processing techniques such as OpenCV function might be another option. These improvements will enable chromosome analysis using a computer, without human error or subjectivity.

Here we started chromosome analysis using CNN-based image recognition models with three limited patterns found in WT and *CTF18*^*-/-*^ TK6 cells. However, they do not cover all chromosome patterns. For example, chromosomes from WAPL-depleted cells have tight and twisted shapes, whereas cohesin-depleted cells have completely separated sister chromatids^1,8^. Moreover, chromosomes lacking primary constrictions are frequently found in Robert syndrome patients who have mutation in *ESCO2* genes^26^. Whether the models distinguish these chromosomes is currently unknown. Moreover, analyzing whether the models can be used to classify the chromosome from other species is important. Future studies can address these tasks for CNN-based models to replace manual analysis conducted by researchers.

## Materials and methods

### Chromosome preparation

Chromosome preparation was performed, as previously described, with a small modification^27^. Metaphase cells were enriched by a treatment with colcemid, a microtubule polymerization inhibitor, for 2 h. Cells were then swollen with 0.075 M KCl and fixed with methanol: acetic acid = 3:1. After dropping on a glass plate and staining with Giemsa solution, the chromosomes were visible under a microscope.

### Image acquisition and cropping

Chromosome images were collected using a Visualix STD1 camera (Visualix) mounted on an inverted microscope (ECLIPSE Ni; Nikon) with a 100 × NA 1.49 objective lens. Subsequent image cropping was performed using the ImageJ software.

### Preprocessing of input data

The original cropped chromosome images were resized to 224 × 224 pixels to match the image size of each pretraining model. The jpg images were converted into tensor images. In addition, if there were other objects in the image, such as other chromosomes thought to negatively affect model learning, they were replaced with white (255, 255, 255). To highlight the shape of the chromosomes, the brightness, contrast, and gamma were adjusted. The difference in staining shade between each image was reduced by fixing the average and standard deviation of each separated RGB channel (for the three colors red, green, and blue). These processes were applied to all chromosome images. The values of the average and standard deviation in normalization were 64 and 16, respectively. The equation $dst\left( {x,y} \right)$ used to highlight the shape of chromosomes is $src\left( {x,y} \right)$, which is the pixel value at position $\left( {x,y} \right)$ in the image. Equations (1 and 2) were used to calculate the contrast $\alpha$, brightness $\beta$, and gamma correction $\gamma$. In this study, we set $\alpha = 3.0$,$\beta = 80.0, and \gamma = 3.0$.

1 $$dst\left( {x,y} \right) = \alpha\,src\left( {x,y} \right) + \beta$$

2 $$y = \left( \frac{x}{255} \right)^{\gamma } \times 255$$

### Plasmid construction and transfection

*CTF18* KO-Puro and *CTF18* KO-Bsr were generated from genomic PCR products combined with puromycin or blasticidin S selection marker cassettes. Genomic DNA sequences were amplified using the primers 5’- GCGAATTGGGTACCGGGCCCactgcctctgggtggatgagtttg -3’ and 5’- CTGGGCTCGAGGGGGGGCCgtgccacctgcagcccaggtagatg -3’ (for the left arm of the KO construct); and 5’- TGGGAAGCTTGTCGACTTAAgtgagtgatgtgaggtccgtctctg -3’ and 5’- CACTAGTAGGCGCGCCTTAAccggctgtacaggaactagacatagg -3’ (for the right arm of the KO construct). The amplified PCR products were purified by gel extraction and cloned into DT-Ap/Puro or DT-Ap/Bsr vectors digested with ApaI and AflII using the GeneArt Seamless Cloning and Assembly kit (Thermo Fisher Scientific). A gRNA to introduce a DSB into the *CTF18* locus was designed using CRISPR direct (https://crispr.dbcls.jp/). Two phosphorylated oligo DNAs, 5’- CACCGgcgtcacgcggggtactctg-3’　and 5’- AAACcagagtaccccgcgtgacgcC-3’, were annealed and ligated with px330 cut by BbsI. *CTF18* knockout vectors were then co-transfected with the gRNA expression vector into TK6 cells using Neon Transfection System (Thermo Fisher Scientific).

### Western blotting analysis

Western blotting was performed, as previously described^27^, using antibodies against CTF18 (Santa Cruz Biotechnology) and *β*-actin (Proteintech), followed by incubation with horseradish peroxidase-conjugated anti-mouse IgG secondary antibody (Cell Signaling Technology). Proteins were visualized using ImmunoStar LD according to the manufacturer’s protocol.

### Growth curve

WT TK6 and *CTF18*^*-/-*^ TK6 cells were cultured at 37 °C in RPMI medium (Wako) supplemented with 5% house serum (Gibco), penicillin/streptomycin mix (Nacalai Tesque), 2 mM l-glutamine (Nacalai Tesque), and 100 μM sodium pyruvate. To plot growth curves, each cell line was cultured in three different wells of 24 well-plates and passaged every 24 h. Cell numbers were determined using flow cytometry. 15 μl of cell suspension was analyzed, and viable cells determined by forward scatter and side scatter were counted.

### Visualizing the basis for classification decisions using Grad-CAM

Grad-CAM, proposed by Selvaraju et al*.*^20^, uses the gradient of the classification score for the convolutional features determined by the network to provide a visual indication of the parts of the image that are most important for classification.

## Supplementary Information

Supplementary Information.

## Data Availability

The research data set files are available at Mendeley Data (https://doi.org/10.17632/jtfbcft2r5.1). Code for the models used in this study is available at https://github.com/ikmtd/Classification_Demonstration.git.
