# Supplementary material for: Application of neural network-based image analysis to detect sister chromatid cohesion defects
Source: Sci Rep. 2023 Feb 6;13:2133. doi: 10.1038/s41598-023-28742-6 (PMC9902603; doi:10.1038/s41598-023-28742-6)
Supplement: Supplementary file 1 — Supplementary Information. [file 41598_2023_28742_MOESM1_ESM.pptx]

## Slide 1
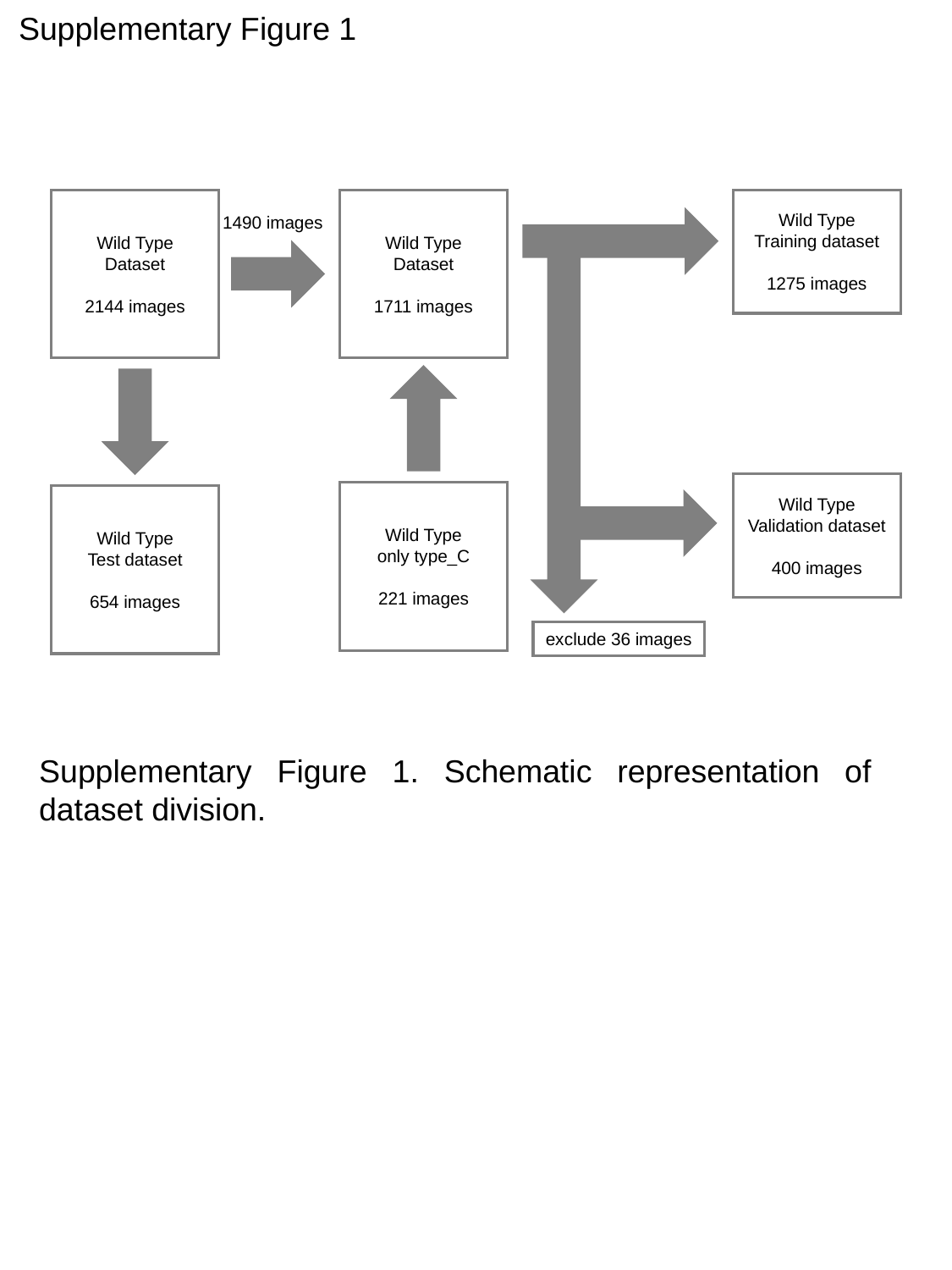

Supplementary Figure 1
Wild Type
Dataset
2144 images
Wild Type
Dataset
1711 images
Wild Type
Training dataset
1275 images
1490 images
Wild Type
Validation dataset
400 images
Wild Type
only type_C
221 images
Wild Type
Test dataset
654 images
exclude 36 images
Supplementary Figure 1. Schematic representation of dataset division.

## Slide 2
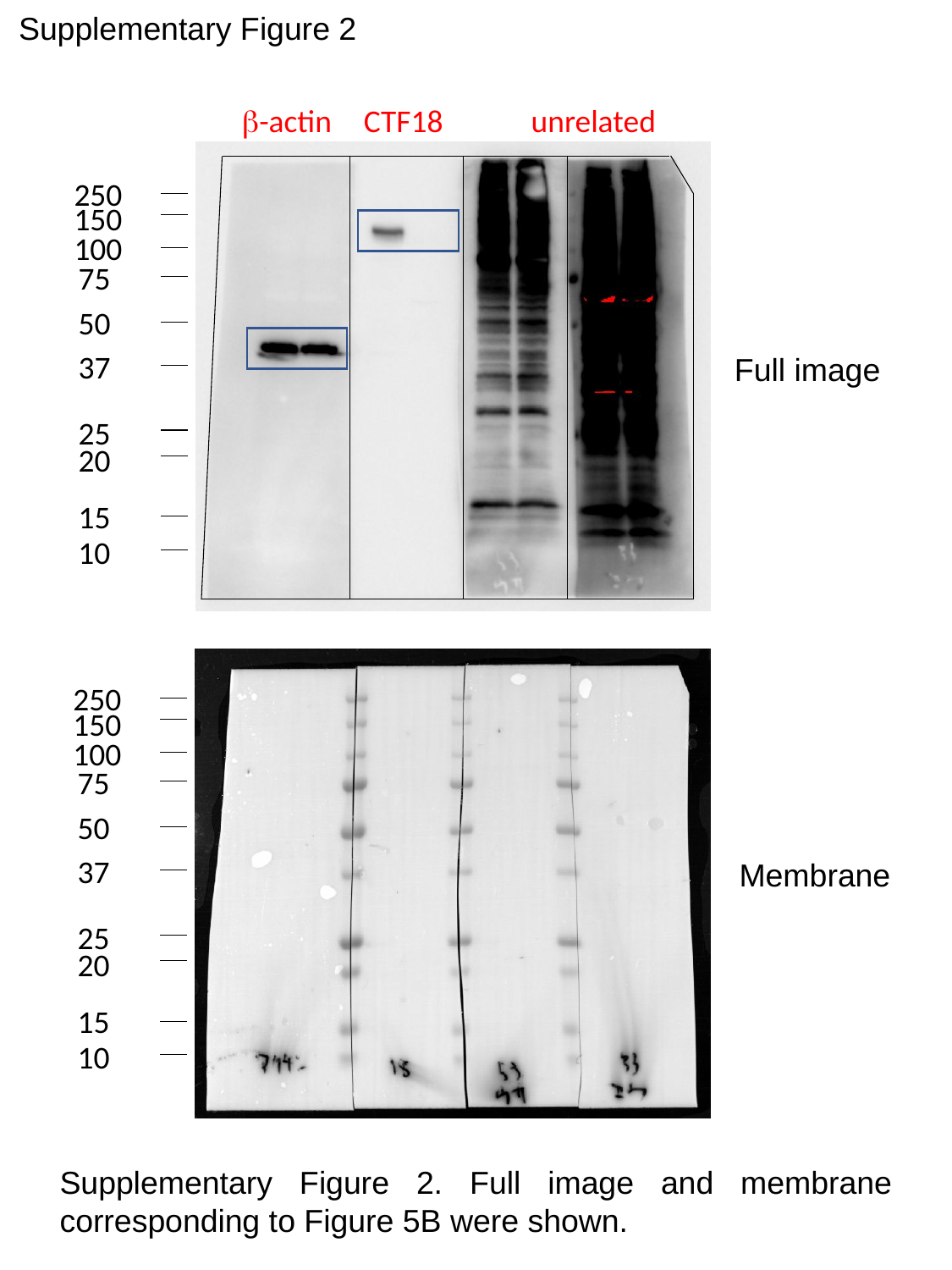

Supplementary Figure 2
b-actin
CTF18
unrelated
250
150
100
75
50
37
25
20
15
10
Full image
250
150
100
75
50
37
25
20
15
10
Membrane
Supplementary Figure 2. Full image and membrane corresponding to Figure 5B were shown.

## Slide 3
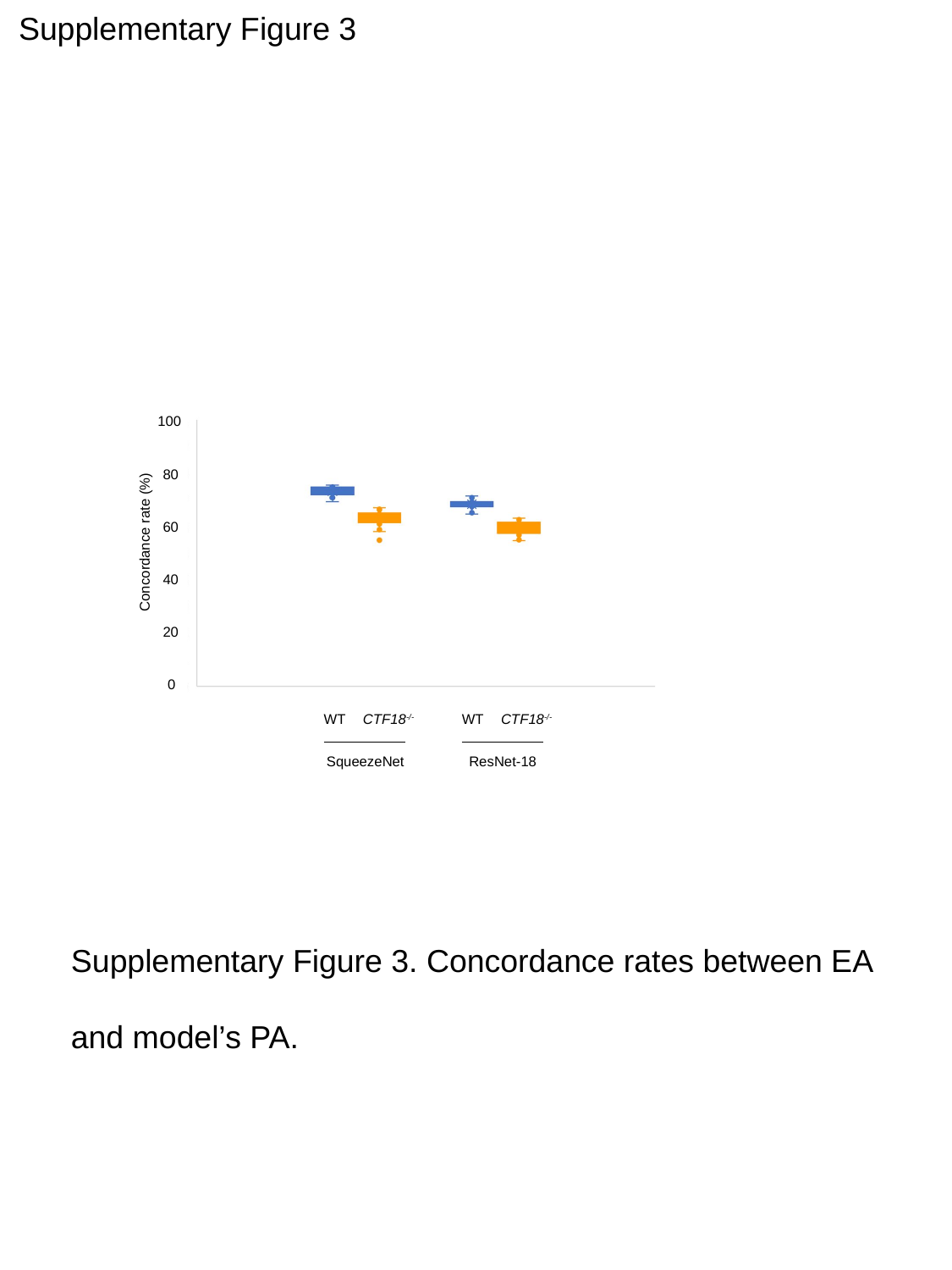

Supplementary Figure 3
100
80
60
Concordance rate (%)
40
20
0
WT
CTF18-/-
WT
CTF18-/-
SqueezeNet
ResNet-18
Supplementary Figure 3. Concordance rates between EA and model’s PA.

## Slide 4
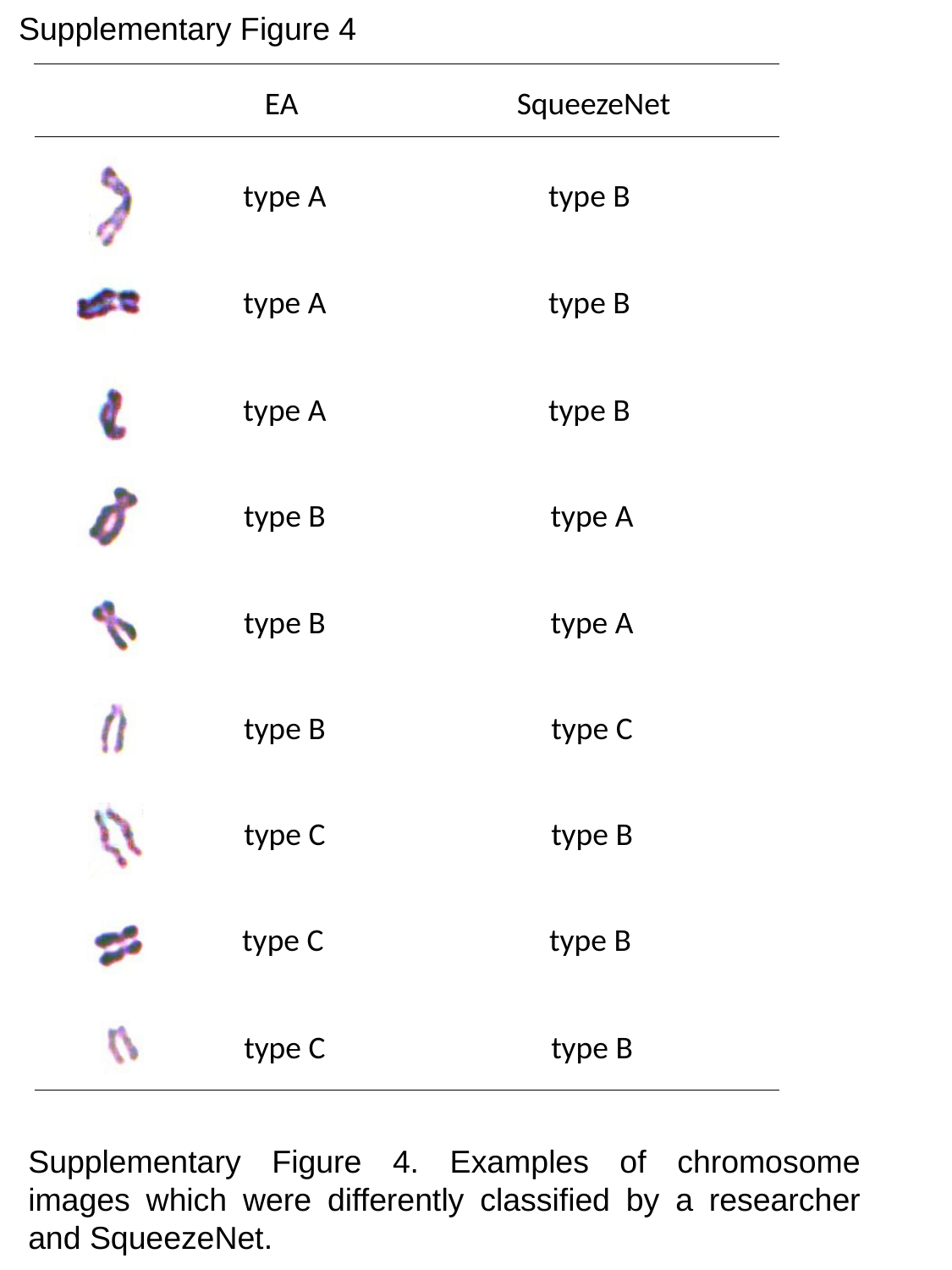

Supplementary Figure 4
EA
SqueezeNet
type A
type B
type A
type B
type A
type B
type B
type A
type B
type A
type B
type C
type C
type B
type C
type B
type C
type B
Supplementary Figure 4. Examples of chromosome images which were differently classified by a researcher and SqueezeNet.
